# Targeted gene delivery in tumor xenografts by the combination of ultrasound-targeted microbubble destruction and polyethylenimine to inhibit survivin gene expression and induce apoptosis

**DOI:** 10.1186/1756-9966-29-152

**Published:** 2010-11-23

**Authors:** Zhi-Yi Chen, Kun Liang, Ri-Xiang Qiu

**Affiliations:** 1 Department of Medical Ultrasound, The Third Affiliated Hospital of Guangzhou Medical University, Guangzhou 510150, PR China; 2 Department of Obstetrics and Gynecology, Guangzhou Research Institute of Obstetrics and Gynecology, The Third Affiliated Hospital of Guangzhou Medical University, Guangzhou 510150, PR China; 3 Department of Pharmacy, The Third Affiliated Hospital of Guangzhou Medical University, Guangzhou 510150, PR China

## Abstract

**Background:**

Noninvasive and tissue-specific technologies of gene transfection would be valuable in clinical gene therapy. This present study was designed to determine whether it could enhance gene transfection *in vivo *by the combination of ultrasound-targeted microbubble destruction (UTMD) with polyethylenimine (PEI) in tumor xenografts, and illuminate the effects of gene silencing and apoptosis induction with short hairpin RNA (shRNA) interference therapy targeting human survivin by this novel technique.

**Methods:**

Two different expression vectors (pCMV-LUC and pSIREN) were incubated with PEI to prepare cationic complexes (PEI/DNA) and confirmed by the gel retardation assay. Human cervical carcinoma (Hela) tumors were planted subcutaneously in both flanks of nude mice. Tumor-bearing mice were administered by tail vein with PBS, plasmid, plasmid and SonoVue microbubble, PEI/DNA and SonoVue microbubble. One tumor was exposed to ultrasound irradiation, while the other served as control. The feasibility of targeted delivery and tissue specificity facilitated by UTMD and PEI were investigated. Moreover, immunohistochemistry analyses about gene silencing and apoptosis induction were detected.

**Results:**

Electrophoresis experiment revealed that PEI could condense DNA efficiently. The application of UTMD significantly increases the tissue transfection. Both expression vectors showed that gene expressions were present in all sections of tumors that received ultrasound exposure but not in control tumors. More importantly, the increases in transgene expression were related to UTMD with the presence of PEI significantly. Silencing of the survivin gene could induce apoptosis effectively by downregulating survivin and bcl-2 expression, also cause up-regulation of bax and caspase-3 expression.

**Conclusions:**

This noninvasive, novel combination of UTMD with PEI could enhance targeted gene delivery and gene expression in tumor xenografts at intravenous administration effectively without causing any apparently adverse effect, and might be a promising candidate for gene therapy. Silencing of survivin gene expression with shRNA could be facilitated by this non-viral technique, and lead to significant cell apoptosis.

## Introduction

Gene therapy holds great promise for the treatment of cancer diseases. Successful gene therapy requires safe and efficient delivery systems [1]. Most viral vectors pose a potential risk of insertional mutagenesis and interference responses [2]. Nonviral delivery systems are safe and easy to apply, but suffer from low transfection efficiency and transient gene expression [3]. Although methods such as cationic polymers could enhance the gene transfection *in vitro *[1], the results of *in vivo *studies were still not so satisfactory because targeting vectors have to overcome chemical and structural barriers to reach cells [4]. Therefore, non-viral gene transfer has low efficiency *in vivo *and transfection with intravenously administered plasmid DNA is difficult [5]. More recently, in order to elevate the transfection efficiency of non-viral vector system, microbubble and the sonoporation inducted by ultrasound could be used to increase the uptake of plasmid DNA targetedly [6-9]. Ultrasound-targeted microbubble destruction (UTMD), as a means of stimulating cell membrane permeabilisation for the purposes of transferring plasmid DNA or drug into cells, has offered advantage over viral technologies [10-12]. When UTMD was combined with cationic polymers or liposome, the gene transfection efficiency had been markedly improved [4,11,13-16]. However, most studies with this technology have mainly used reporter gene to show transfection rather than efficacy in cancer gene therapy.

Survivin, the smallest member of the mammalian inhibitors of the apoptosis protein (IAP) family [17,18], is upregulated in various malignancies to protect cells from apoptosis [18,19], which justifies its role as a rational target for cancer therapy [20]. RNA interference (RNAi) is a potent and convenient technique, and is widely used in the applications such as gene function analysis [7,21,22]. RNAi mediated survivin knock-down in different cell lines caused increased apoptosis rates and cell cycle arrest, reduced viability and clonogenic survival as well as chemosensitization and radiosensitization [20,23,24]. In contrast to chemically synthesized, sequence-specific double-stranded short interference RNA (siRNA), short-hairpin RNA (shRNA) expression vectors could be used to establish stable gene expression, and could be a powerful tool for anticancer therapy [21,22]. Apoptosis induction by shRNA targeting survivin represents an efficient, novel strategy for cancer gene therapy [25-27]. These shRNA expression vectors could be deliveried by UTMD systems, but related study was rare [28].

For this purpose, in this present study, gene transfer of tumor xenografts in nude mice was performed through intravenous injection using the method of the combination of UTMD and polyethylenimine (PEI). We also tested the effects of gene silencing and apoptosis induction with shRNA interference therapy targeting human survivin by this novel technique. The result showed that, transfection efficiency was significantly improved and provided a new way for *in vivo *cancer gene therapy.

## Materials and methods

### Preparation of Plasmid DNA

pCMV-LUC (7.4 kb) was constructed by cloning the luciferase gene from the pGL3-Promoter Vector (5.01 kb, Promega Corp., Madison, WI, USA) into pcDNA3.1 (5.42 kb, Invitrogen, San Diego, CA, USA) at the *Bam *HI and *Hind *III sites [29].

The pSIREN-DNR-DsRed-Express Vector (6,7 kb, BD Biosciences Clontech, USA), was an expression vector for red fluorescence protein (RFP) gene, which excitation and emission maxima occur at 557 nm and 579 nm, respectively. A shRNA expression vector targeting human survivin gene (GenBank accession no. NM_001168) was designed and synthesized as described previously [28]. The selected reconstructed plasmid for transfection was extracted and purified using a Qiaquick Kit (Qiagen, Crawley, UK). The double strand oligos generating survivin shRNA were subcloned into linearized expression vector at the *Bam *HI and *EcoR *I sites. The specific recombinant shRNA vector was named pSIREN-S. Similarly, a non-specific control vector was constructed, which was named pSIREN-C.

The concentration of isolated plasmid DNA was determined by absorbance at 260 nm wavelength (A260) using UV spectrophotometry (DU-640, Beckman Coulter, Fullerton, CA, USA) and resuspended to a final concentration of 1 μg/μl in buffer. In addition, the absorbance ratio of the A 260 to A 280 was between 1.8 and 2.0, indicating that the purified plasmid DNA was free of contaminants. The recombinant plasmid was evaluated by Bio Imaging Systems (Syngene, Synoptics Ltd, Cambridge, UK).

### Preparation of Transfection Complexes

Branched PEI with an average molecular weight of 25 kDa was obtained from Sigma-Aldrich (St. Louis, MO, USA). An aqueous stock solution of PEI was prepared by diluting 1 mg of the commercial solution in 1000 ml DI water, neutralized with HCl and filtering at 0.2 μm (Millipore, Bedford, MA, USA). Two PEI/DNA complexes were performed by mixing PEI and plasmids at 1:4 to 8:1 of N/P ratio [PEI nitrogen: DNA phosphate ratio, based on the recognition that 1 μl of PEI stock solution contains 10 nmol of amine nitrogen and 1 μg of DNA contains 3 nmol of phosphate [30]]. The complexes incubated for 20-30 min at room temperature and stored in 4°C. Electrophoresis was carried out for 40 min at 80 V. The separations were visualized to determine the optimal ratio of PEI/DNA complexes.

The suspension of SonoVue microbubbles (Bracco Research, Switzerland) were reconstituted before use by injecting 5 mL of 0.9% saline solution. Before the experiments, plasmid DNA (30 μg) or PEI/DNA complexes and SonoVue microbubble (100 μL) were gently agitated with phosphate buffered saline (PBS) to a final volume of 200 μL to prepare the transfection complexes (P/SonoVue and P/SonoVue/PEI, P indicated as plasmid) as detailed previously [11]. All the complexes were prepared by incubation for 15 min at room temperature.

### Cell Culture

Human cervical cancer cell lines (HeLa) were obtained from China Center for Type Culture Collection (CCTCC) and incubated in Dulbecco's modified Eagle's medium (DMEM, Gibco, USA) with 10% fetal bovine serum (FBS, Gibco, USA) and 100 U/ml penicillin, 100 μg/ml streptomycin, at 37°C in a humidified environment of 5% CO_2 _and 95% air. Total cell count was determined with a hemocytometer (Burker Turk). Initial cell viability was determined by means of exclusion with trypan blue dye (Sigma-Aldrich, USA). Exponentially growing cells were used in all experiments. Before animal modeling, Hela cells were harvested, collected and centrifuged, and then resuspended in 100 μl DMEM to prepare single cell suspension.

### Animal Protocol

Female Balb/c (nu/nu) mice, 4-6 week old, weighing 15-21 g, were purchased from experimental animal research center. All the mice were treated and housed according to approved guidelines (Guidelines for the Care and Use of Laboratory Animals).

The mice were fixed on superclean bench according to the principle of aseptic operation, and inoculated subcutaneously into the flank with 2 × 10^6 ^cells per mouse after local sterilized. The mice were continued to be raised at specified pathogen free (SPF) qualification after operation, being observed one time every two days. Two weeks later, the experiments were initiated when the tumors reached a size of 5-10 mm.

### Experimental Grouping of Gene Delivery

To analyze the impact of the combination of UTMD and PEI on the RFP expression, nude mice bearing tumor xenografts were selected, randomly divided into four groups, four mice each group: A group: PBS group (negative control); B group: naked pSIREN-C group; C group: pSIREN-C/SonoVue group; D group: pSIREN-C/SonoVue/PEI group.

To investigate the effect of UTMD combined with PEI on the luciferase activity, another 20 nude mice were selected, randomly divided into five groups, four mice each group, a group; PBS group (negative control); b group: naked pCMV-LUC group; c group: pCMV-LUC/SonoVue group; d group: pCMV-LUC/SonoVue/PEI group; e group: after the injection of pCMV-LUC/SonoVue/PEI complexes, the tumor xenografts were not received ultrasound irradiation and compared with group d to understand the impacts of this transfection method and ultrasound irradiation on other non-target organs (livers, kidneys, lungs, hearts). In other groups, only one side of the tumor xenografts was received irradiation, while the other served as control.

The total dose of injection was 200 μl, and the plasmid dosage was 30 μg/mouse. The microbubbles were mixed with plasmid solution or PEI/DNA complex at the proportion of 1:1. All the plasmid DNA or complexes were administrated by tail vein. The mice were anesthetized by diethylether and fixed on the flats. The tumor xenografts were subsequently sonicated by a transducer (Accusonic, Metron Medical Australia Pty. Ltd.) placed on the skin with contact gel (Aquasonic 100, Parker Laboratories Inc., USA). Ultrasound parameters were set at 3 MHz, 2 W/cm^2^, 2 min, duty cycle 20%. During the exposure, the ultrasound transducer was moved around in a circular motion to ensure the whole tumor xenograft exposed.

### Analysis of Transfection Efficiency

3 days after ultrasound treatment [9], nude mice were sacrificed by cervical dislocation. The specimens of tumor xenografts, the skins around the tumors, hearts, livers and lungs, were immediately harvested, embedded in optimal cutting temperature compound (OCT, Tissue-Tek, Sakura Finetek, Torrance, CA, USA), and stored at -80°C until further analyses. Cross sections (10 μm-thick slices) were cut with a cryostat (CM1900, Leica, Germany) and affixed to glass slides. Fluorescence expression and distribution pattern were observed with confocal laser microscopy (Fluoview FV500, Olympus, Japan). Digital image subtraction method was devised to eliminate autofluorescence. Slices were coded so that analyses were performed without knowledge of which treatment each individual animal had received. For each sample, RFP expression and transfection efficiency were evaluated in six randomly chosen fields per section.

For examination of luciferase reporter gene expression, tumor xenografts and the non-targeted organs in group d and e were removed and homogenized, frozen in liquid nitrogen, and stored at -80°C. Luciferase activity in the tissue lysate was measured using a Lumat LB9507 instrument (Berthold, Bad Wildbad, Germany). Luciferase background (100-200 RLU) was subtracted from each value and transfection efficacy is expressed as RLU/organ or RLU/tumor [31]. One million RLU correspond approximately to 2 ng luciferase.

### Gene Silencing and Apoptosis Induction Effects of shRNA Expression Vector Targeting Survivin Transfected by UTMD and PEI

A total of 18 mice were randomly divided into 3 experimental groups, 6 mice each group. Control group, mice were received injections of PBS; pSIREN-S +UTMD group, mice were received injections of pSIREN-S/SonoVue and followed by local ultrasound irradiation; pSIREN-S + UTMD + PEI group, mice were received injections of pSIREN-S/SonoVue/PEI complexes and followed by local ultrasound irradiation. All injections were performed with the plasmid DNA dose of 30 μg/mouse. The number of dead mice was noted every day. 21 days after injection, the tumor-bearing mice were humanely sacrificed and the solid tumors were harvested.

### Immunohistochemistry

The samples were fixed with formaldehyde, dehydrated with a graded alcohol series, and embedded in paraffin. The sections were incubated with primary antibodies against survivin, bcl-2, bax and caspase-3 (1:100 dilution, Santa Cruz Biotechnology) and then incubated with appropriate biotinylated secondary antibody as detailed previously [32]. The colorimetric detection was performed by using a DAB detection kit (Boster Biological Technology Co. Ltd., Wuhan, China). Images were acquired with a microscope (BX51, Olympus, Japan). The assessment of the immunohistochemical results were modified from that described previously [33,34]. The percentage of cells expressing the marker were classified qualitatively based on the intensity of staining and the percent of cells as follows: score 1: no reactivity; score 2: low intensity staining in less than 10% of cells; score 3: low to moderate intensity staining in 11% to 30%; score 4: moderate to strong staining in 31% to 50%; score 5: diffuse, strong intensity staining in 50%.

### Histopathology

Serial sections of tumor tissue were processed for routine histological examination. The specimens were washed with PBS to remove blood, fixed with formaldehyde, dehydrated with a graded alcohol series, and embedded in paraffin. Hematoxylin eosin staining (H&E) was performed on the specimens, for histopathologic evaluation of hemorrhage, necrosis, and inflammation.

### Statistical Analysis

Statistical analyses were performed by the SPSS 13.0 software package (SPSS, Inc, Chicago, IL). All values were expressed as mean ± SD. Analysis of variance with *t *test and analysis of variance (ANOVA) test were used to determine the significance of the difference in a multiple comparison. If the ANOVA was significant, the Tukey's procedure was used as a post hoc test. Differences with a *P *value of less than 0.05 were considered to be statistically significant.

## Results

### Identification of pCMV-LUC by Restriction Enzymes Digestion

**Figure 1:**
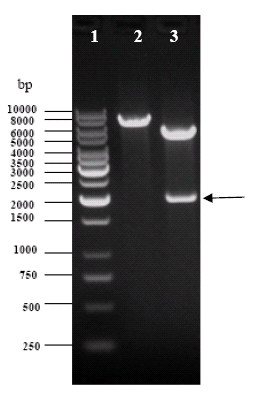
**Identification of pCMV-LUC by restriction enzymes digestion**. 1, Marker, 1 kb ladder; 2, pCMV-LUC; 3, pCMV-LUC/*Hind *III + *Bam *HI; the plasmid pCMV-LUC or with the restriction enzymes digestion showed two bands after electrophoresis. A correct insertion was showed a band of 2000 bp (as arrowhead indicated) cut off by *Hind *III and *Bam *HI.

After double-enzyme cutting by *Bam *HI and *Hind *III, the restriction enzymes digestion results showed that the objective fragment of the pCMV-LUC plasmid could be detected at around 2000 bp, which was exactly coincidence with the size of the designed DNA (Figure 1).

### Gel Retardation Analysis of PEI/DNA Complexes

**Figure 2:**
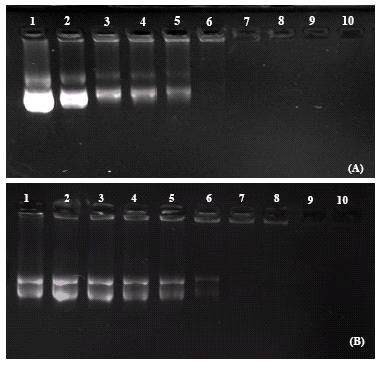
**Electrophoretic patterns of plasmid DNA complexes prepared with PEI at various N/P ratios: N/P ratio = PEI nitrogen/DNA phosphate; (A) pCMV-LUC, (B) pSIREN-S**. Lanes 1-10: the N/P molar ratios of 1/4, 1/2, 1, 3/2, 2, 5/2, 3, 4, 6, and 8.

Agarose gel electrophoresis analysis showed that (Figure 2), with the increase of N/P ratio of PEI/DNA complexes, the plasmid DNA migrated more slowly. When N/P ≥ 3, the plasmid DNA migration could not be observed, and the PEI/DNA complexes with positive charge remained in the hole. PEI could effectively condensate the plasmid DNA, and the electrophoresis analyses of both plasmids were similar (Figure 2A-B). According to the results of electrophoresis, the N/P ratio was chose for 6 in this study and used in the following experiments.

### Enhanced RFP Expression in Transplanted Tumors by Combination of UTMD and PEI

**Figure 3:**
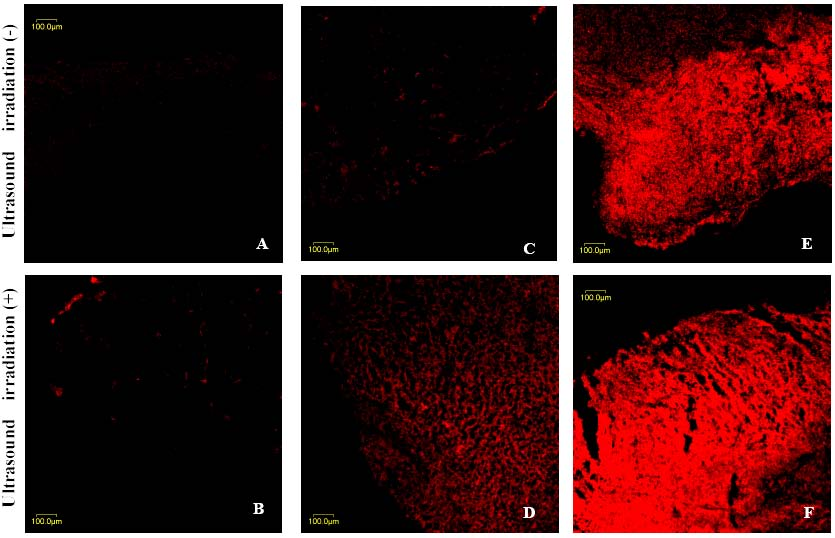
**Fluorescent microphotographs of the tumor xenografts in nude mice after intravenous injection of naked pSIREN-C (A, B), pSIREN-C/SonoVue complex (C, D) and pSIREN-C/SonoVue/PEI complex (E, F) with or without ultrasound irradiation**. Ultrasound irradiation parameters were as follow, irradiation time = 2 min, intensity = 2 W/cm^2^, frequency = 3 MHz, and duty cycle = 20%. UTMD = ultrasound targeted microbubble destruction; PEI = polyethylenimine; bar = 100 μm.

Regardless of ultrasound irradiation, there was no obvious RFP expression in Group A and B (Figure 3A-B). Without ultrasound irradiation, there were only a few cells expressing RFP in pSIREN-C/SonoVue group and red fluorescent signal was weak in the majority of samples (Figure 3C). However, the RFP expression was enhanced and increased when the injection of plasmid DNA and SonoVue combined with ultrasound irradiation (Figure 3D), but it was still weaker than that of pSIREN-C/SonoVue/PEI group (Figure 3E). When UTMD combined with PEI, RFP expression was increased significantly with strong density and signal (Figure 3F).

### Enhanced Luciferase Activity by Combination of UTMD and PEI

**Figure 4:**
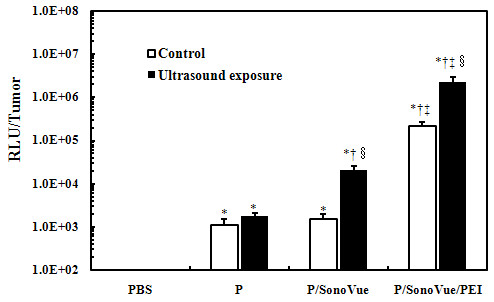
**Luciferase expressions of tumor xenografts in nude mice with UTMD and PEI**. Control: non ultrasound exposure; P: pCMV-LUC; in the same condition (control or ultrasound exposure), as compared with PBS group, *P < 0.01; as compared with P group, †*P *< 0.01; as compared with P/SonoVue group, ^‡^*P *< 0.01; as compared with control group, ^§^*P *< 0.01.

The luciferase expression could not be increased by ultrasound irradiation after the injection of naked plasmid (*t *= -2.174, *P=*0.095, Figure 4). Without ultrasound exposure, microbubble could not significantly improve the luciferase activity of tumor tissues. But the application of UTMD could significantly promote the transfection efficiency (*t *= -11.433, *P *< 0.01), with the luciferase expression increased by about 14 fold.

The transfection efficiency was the highest when UTMD combined with PEI. As compared with non-irradiated tumor, the luciferase activity of irradiated samples has increased by about 10 fold (*t *= -11.633, *P *< 0.01). And the luciferase expression increased by about 111 fold when compared with that of non-combined PEI group (*P *< 0.01). This demonstrated that the combination of UTMD with PEI would significantly facilitate the transfection efficiency.

### Analysis of Tissue Targeting

**Figure 5:**
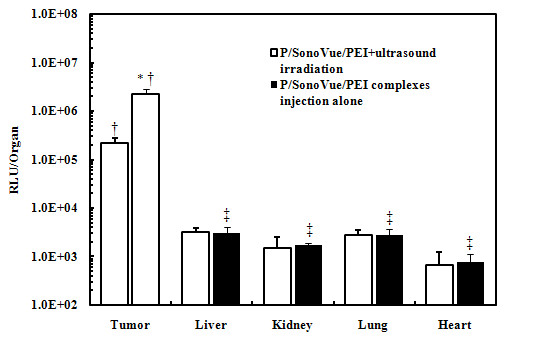
**Luciferase expressions of non-target organs in nude mice with UTMD and PEI**. P: pCMV-LUC; as compared with non-irradiated tumors, *P < 0.01; as compared with other organs, ^†^*P *< 0.01; as compared with P/SonoVue/PEI complexes injection alone, ^‡^*P *> 0.05.

As shown in Figure 5, when the tumor xenografts was irradiated (group d), the increase extent of luciferase activity was significantly higher than that of non-irradiated tumor and other tissues and organs (all *P *< 0.01). Livers, lungs, kidneys and hearts in group d, e, had relative low luciferase activity level, but all were lower than that of the tumor xenografts (*P *< 0.01). The ultrasound irradiation of the transplanted tumors had no evident impact on other organs (*P *> 0.05).

### Degradation of Survivin Was the Main Trigger for Apoptosis Induction in Nude Mice

**Figure 6:**
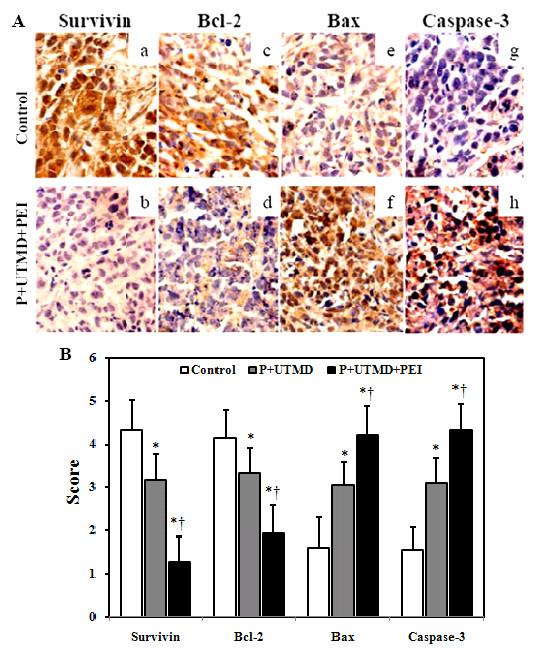
**Apoptosis induction by downregulation of survivin in nude mice**. (A) P: pSIREN-S; Representative expressions of survivin (a and b), bcl-2 (c and d), bax (e and f) and caspase-3 (g and h) protein were shown. Positive expressions in serial sections were shown in representative photomicrographs (positive stain was brown). Magnification = 400×. (B) The scores were classified as 1 to 5, based on the intensity of staining and the percent of positive expression cells. The results indicated that inhibition of survivin by administration of shRNA plasmid by UTMD technique resulted in apoptosis induction by downregulating bcl-2 and survivin expression, and upregulating the activity of caspases-3 and bax. Furthermore, the combination of UTMD and PEI could lead to the most significant gene downregulation and cell apoptosis. *P < 0.001 vs control, †*P *< 0.001 vs P+UTMD group.

As shown in Figure 6A(a-b) and 6B, the microscopic examination of stained tumor sections showed that the score of survivin expression in control group and pSIREN-S +UTMD group were significantly higher than that of pSIREN-S + UTMD + PEI group (both *P *< 0.01). Comparing with that of control and pSIREN-S + UTMD group, the score of bcl-2 protein expressions in pSIREN-S + UTMD + PEI group also resulted in downregulation markedly (both *P *< 0.01, Figure 6A(c-d) and 6B). Moreover, As shown in Figure B, score of bax [Figure 6A(e-f)] and caspase-3 [Figure 6A(g-h)] protein expressions in pSIREN-S + UTMD + PEI group was upregulated remarkably as comparing with control group and pSIREN-S + UTMD group (all *P *< 0.01, Figure 6B).

### Histology Examination

In pSIREN-S + UTMD + PEI group, H&E staining showed that the integrities of tumor xenografts were good. The histologic structure of livers, kidneys, lungs, hearts and other organs were normal, and no necrosis or fibrosis changes were seen. Moreover, the results showed no abnormalities such as inflammation or degeneration in any tissues.

## Discussion

PEI, as one of the most effective poly-cationic gene vectors, could condense plasmids DNA into cationic polymers, protect the plasmids against being degraded by nucleinase or enzymes within a few hours, and enhance the endocytosis of plasmids DNA, thus promoting gene transfection *in vivo *[31,35]. On the other hand, ultrasound could increase transfection efficiency *in vivo *and *in vitro*. Microbubbles could significantly improve the transgenic expression. Moreover, ultrasonic energy could be focused on the target site of gene transfer by local irradiation [11]. It was particularly important for gene transfer in deep tissues. A lot of literature [13-16,36] reported that the combination of cationic polymers and ultrasound could improve transfection efficiency. Lawrie *et al. *[13] reported that UTMD enhanced approximately 300 fold increments in transgene expression after naked DNA transfection. While UTMD and polyplex yielded transgene expression levels approximately 3000 fold higher than after naked DNA alone. Anwer *et al. *[14] reported that UTMD resulted in a significant increase in gene transfer to the tumors by the tail vein injection and the maximum of expression was 270-fold. In this study, when the application of UTMD combined with PEI, the transfection efficiency for both plasmids in the tumor xenografts could be significantly improved, providing a new strategy for cancer gene therapy.

UTMD could facilitate targeted gene therapy, thus significantly enhance gene transfection *in vivo*. The results of our study showed that, after intravenous injection of plasmids DNA, there was obvious gene expression in the irradiated tumors. And the difference had statistical significance when compared with that of non-irradiated tumors. Similar to our study, Haag *et al. *[37] established two tumors in each animal, injected the ODN-loaded microbubbles intravenously, and then exposed only one of the tumors to ultrasound. Their results showed that, digoxigenin staining intensity was significantly stronger in treated tumors (16-49%) that were exposed to ultrasound as compared with the untreated collateral control tumors (2-18%). Dittmar *et al. *[38] applied pulsed high intensity focused ultrasound to expose one tumor while the other tumor served as a control and found that local exposure in tumors could enhance expression of green fluorescent protein (GFP). Moreover, UTMD could transduce plasmids into target tissue when systemic administration rather than direct target organ delivery by catheter-based approaches or operative injection. And this was particularly important in cardiovascular as well as gene therapy of inaccessible tumors. Howard *et al. *[39] reported that, systemic delivery of Ad-GFP microbubbles pretreated with complement and injected in the tail vein of nude mice resulted in high level of transgene within the tumor alone. Both fluorescence microscopy and GFP immunohistochemistry demonstrated UTMD induced specific transduction in the targeted cells only, with no uptake in hearts, lungs or liver. Chen *et al. *[2] incorporated plasmids into the phospholipid shell of gas-filled microbubbles, which were then infused into rats and destroyed within the pancreatic microcirculation with UTMD technology. They found that UTMD allowed relatively noninvasive delivery of genes to pancreatic islets with efficiency sufficient to modulate the function of β-cell, and a low level of luciferase activity was detected in all organs within the ultrasound beam. Activity of skeletal muscle or right kidney which lie outside the ultrasound beam was not detected in their study. This data illustrated that this technique largely could prevent the problem of hepatic uptake seen with viral vectors. Moreover, study indicated [9] that the reticuloendothelial system was not a limiting factor for the ultrasound-based gene delivery with these experimental conditions. While Huber *et al. *[5] found that, after intratumoral DNA injection, ultrasound induced a 10-fold increase of β-galactosidase positive cells. In contrast, ultrasound was not found to enhance reporter gene expression after intravenous plasmid application. But they did not apply the UTMD technology.

To further enhance the transfection efficiency of UTMD, DNA can be protected by the complexation of cationic polymers and microbubbles. Because both membrane of SonoVue microbubble and plasmid DNA bear a net negative charge [40], the binding of plasmid DNA and microbubbles are likely to be weak and transient. Cationic polymers, such as PEI, have strong capacity to bind to negatively charged DNA and proteins. It was hypothesized that P/PEI complexes were adsorbed to the surface of microbubbles through electrostatic interaction, and P/SonoVue/PEI complexes were formed. The complexes could be released targetedly by ultrasound irradiation. In addition, ultrasound irradiation could enhance gene transfection of tumors as well, and reduce gene expression of other non-target organs. SonoVue microbubbles could significantly increase the transfection efficiency, but further study was still needed to validate the specific mechanisms.

Just like the study of Gao *et al. *[41], 3 MHz ultrasound in our study facilitated the irradiation of superficial tumor xenografts. Ultrasonic energy was more focused, and had no significant impacts on other organs. As the N/P ratio increased, the toxicity will be grater, too [31]. The results indicated that this N/P ratio in our experiment could enhance *in vivo *transfection efficiency effectively. But it was still need to further analysis and different N/P ratio should be compared. In addition, the transfection efficiency is related to the cell line, microbubble components and DNA vectors. Blood supply or reaction to some certain gene was different, the effects would be different. Moreover, tumor growth was very rapid in the cells with higher division rate, and cell proliferation would dilute the effect of transfection. It would lead to elimination of exogenous plasmid DNA from transfected cells [42]. Furthermore, there are lots of differences in the optimal time points among different organs and tissues, the transfection efficiency will differ for different administration ways, too. Therefore, studies of the optimization analysis of different methods of transfection mediated by the combination of UTMD and PEI should be further investigated.

In mammalian cells, apoptosis is modulated by inhibitors of the apoptosis protein (IAP) families. Cancer cells possess defects in apoptotic, with the consequence of increased resistance to cell death. From the human cancer gene therapy perspective, using molecular antagonists of survivin was one approach which was regarded as a predominant strategy in anticancer therapy for enhancing cancer cell death [25-27]. On the other hand, for the potential use of UTMD as a therapeutic gene delivery system, it is critically important to investigate the apoptosis induction under actual physiological conditions. Diverse molecular mechanisms have been implicated in the apoptosis induction [43,44]. The bcl-2 family of proteins regulates various steps in apoptosis and blocks cell death, whereas pro-apoptotic proteins such as bax promote apoptosis [33,34]. Caspase-3 is the ultimate executioner caspase that is essential for the nuclear changes associated with apoptosis [45]. Moreover, survivin is known to directly or indirectly interact with caspase-3 and subsequently inhibit its activity. In our study, microscopic examination and scoring showed that protein expressions of bax and caspase-3 were up-regulated in P+PEI+UTMD group as compared with those of control group or P+UTMD group, while protein expressions of survivin and bcl-2 were down-regulated markedly. The data indicated that the inhibition of survivin by administration of shRNA expression vectors with the combination of UTMD and PEI resulted in apoptosis induction in nude mice by downregulating bcl-2 expression and upregulating the activity of bax and caspases-3.

## Conclusions

In summary, UTMD could synergistically promote the development and application of other gene transfer methods *in vivo*. It could be used as a safe and effective non-viral gene delivery system. The combination of UTMD and PEI, which could significantly enhance the gene expression of plasmid DNA in the tumor tissue, was a new method of *in vivo *gene transfer with a good prospect. Survivin downregulation with shRNA expression vector mediated by the UTMD and PEI technique could obviously induce apoptosis *in vivo*. This method will provide a noninvasive, safe, promising candidate for tumor gene delivery. More researches are needed to further the efficient, promising novel technique for cancer gene therapy.

## Competing interests

The authors declare that they have no competing interests.

## Authors' contributions

CZY has carried out the study design, molecular biological work, statistical analyses and drafted the manuscript. LK has contributed in literature research and helped to draft the manuscript. QRX has contributed in animal experiment. All authors read and approved the final manuscript.
